# Evaluation of Surgical Treatment Effect on Idiopathic Normal Pressure Hydrocephalus

**DOI:** 10.3389/fsurg.2022.856357

**Published:** 2022-04-07

**Authors:** Ran Sun, Haibo Ning, Ning Ren, Xiuying Xing, Xuejiao Chen, Guihua Li, Xin Li, Lei Chen

**Affiliations:** ^1^Department of Neurology, Clinical College of Neurology, Neurosurgery and Neurorehabilitation, Tianjin Medical University, Tianjin, China; ^2^Department of Neurology, Affiliated Hospital of Jining Medical University, Jining, China; ^3^Department of Neurology, The Fourth Hospital of Baotou, Baotou, China; ^4^Department of Neurology, Tianjin Huanhu Hospital, Tianjin, China

**Keywords:** idiopathic normal-pressure hydrocephalus, ventriculoperitoneal shunt, long-term efficacy, surgical treatment, evaluation

## Abstract

**Background:**

We aimed to observe the long-term effectiveness and safety of the ventriculoperitoneal (VP) shunt in treating idiopathic normal pressure hydrocephalus (iNPH).

**Methods:**

A total of 65 patients with iNPH were retrospectively analyzed. All the patients were treated with VP shunt and the clinical efficacy was assessed using follow-up questionnaire, the Modified Rankin Scale (mRS), and iNPH grading scale (iNPHGS) after operation.

**Results:**

The mean mRS and iNPHGS scores were 1.18 ± 0.83 points and 2.98 ± 1.96 points, respectively, which were statistically significantly different from those before operation [(2.89 ± 0.92) points and (6.49 ± 2.30) points, respectively]. Besides, the patients were divided into the non-improvement group (*n* = 8, 12.3%), the improvement group (*n* = 16, 24.6%), and the marked improvement group (*n* = 41, 63.1%) based on the preoperative and postoperative mRS scores and the total effective rate of the VP shunt was 87.7%. Next, seven patients with negative cerebrospinal fluid tap test (tap test) received the active VP shunt and the score on walking disorder was 2.71 ± 0.76 points before operation and 1.86 ± 0.90 points after operation, showing a statistically significant difference. Moreover, 12 (18.4%) patients had complications after operation, among whom 5 (7.7%) patients manifested ameliorated symptoms after replacing shunt tube and receiving anti-infection treatment, but 3 (4.6%) patients showed no alleviation following pressure adjustment due to advanced age and multiple complications. Six (9.2%) cases of death were recorded during follow-up and only 1 (1.5%) case of sudden death occurred within 2 weeks after operation. In addition, it was found through more than 5 years of follow-up after operation that 12 out of the 23 (52.2%) patients had a good effect at 5 years after operation, 1 (4.3%) patient had been confined to bed due to advanced age and pulmonary infection, and 1 (4.3%) patient died of pulmonary infection and heart failure.

**Conclusion:**

The VP shunt is effective in treating iNPH and it results in a preferable long-term prognosis.

## Introduction

The typical clinical manifestations of idiopathic normal pressure hydrocephalus (iNPH) include gait disorder, cognitive dysfunction, and urinary incontinence (Hakim–Adams syndrome) (1). Among them, gait disorder is the most common and cognitive dysfunction and urinary incontinence may occur at varying frequencies. iNPH can be detected *via* cranial CT scan or MRI and the cerebrospinal fluid pressure of patients shall be normal (70–200 mm H_2_O, 1 mm H_2_O = 0.00098 kPa) (2, 3). Approximately, 50% of patients with iNPH have Hakim–Adams syndrome and the disease should be definitely diagnosed by combining with other clinical manifestations and examination findings when the patients only have one or two symptoms. With incidence and prevalence rates of 5.5/100,000 and 22/100,000, respectively, iNPH mainly influences the elderly people and serves as a cause of reversible dementia (4, 5). iNPH mostly occurs in the elderly aged ≥ 65 years old and cannot be detected easily in the early stage because of insidious onset and the patients have had disease progression once there are clinical symptoms. Besides, the disease may exacerbate within several months, seriously affecting patients' quality of life. As the population aging aggravates in China, iNPH poses a huge challenge to clinical diagnosis and treatment. Some studies have indicated that the occurrence of iNPH is certainly associated with hypertension, cerebrovascular disease, and Alzheimer's disease (5, 6).

There are three major treatment protocols for iNPH in clinic, namely, conservative treatment, ventriculoperitoneal (VP) shunt, and endoscopic third ventriculostomy. Multiple studies have demonstrated that surgery has an obvious therapeutic effect on iNPH and can improve the prognosis of patients. For patients undergoing conservative observation, their symptoms may aggravate gradually after the stable phase and are seldom relieved spontaneously. Therefore, conservative treatment protocols have not been recommended any more in the recent years (7). Currently, the VP shunt is an important treatment method for iNPH, but its long-term efficacy still remains controversial (8, 9) and its safety needs to be further proved.

A total of 65 patients diagnosed with iNPH in the Neurosurgery Department of Tianjin Huanhu Hospital from April 2014 to April 2019 underwent the VP shunt and favorable clinical efficacy was obtained. The follow-up results in this study are now retrospectively reviewed as follows.

## Materials and Methods

### Clinical Data

All the 65 patients met the diagnostic criteria in the International and Japanese Guidelines for iNPH (5, 10) and the VP shunt was performed. There were 39 males and 26 females aged 29–88 years old, with an average of 61.8 ± 11.8 years old. The course of disease was 6–156 and 21.3 ± 26.3 months on average. No apparent primary cause was found among the patients at the initial visit, while 1–3 symptoms related to gait, urinary incontinence, and cognition were detected. Specifically, 61 (93.8%), 51 (78.5%), and 50 (76.9%) patients had gait disorder, cognitive disorder, and urinary incontinence disorder, respectively, before operation. Cranial CT, MRI, and other imaging examinations indicated hydrocephalus. Preoperative lumbar puncture proved that the cerebrospinal fluid pressure was within the normal range, laboratory test results of cerebrospinal fluid showed no obvious abnormalities, and all the patients had indications for the VP shunt. Besides, 34 (52.3%) patients manifested complications, including 22 (33.8%) cases of primary hypertension, 10 (15.4%) cases of stroke history, 9 (13.8%) cases of diabetes history, 6 (9.2%) cases of coronary heart disease history, 1 (1.5%) case of Parkinson's disease history, and 3 (4.6%) cases of epilepsy. All the patients or their families signed the informed consent of treatment and this study was approved by the Ethics Committee of Tianjin Huanhu Hospital.

### Pre-Operative Assessment

As this study was a retrospective case analysis, scale, imaging, and invasive assessment were performed according to telephonic interview of the patients themselves or their guardians as well as inquiry of the clinical manifestations in medical record system. Specifically, the clinical system score and overall living ability were evaluated using iNPH grading scale (iNPHGS) and the Modified Rankin Scale (mRS), respectively. The indicators of clinical results were evaluated by reference to related iNPH guideline and commonly used international evaluation criteria (11). (1) The scale assessment involved gait, cognition, and urinary incontinence assessment. (a) Gait assessment (12): The gait was scored (0–4 points) based on the autonomy and whether assistance was needed during walking in usual, as follows: zero point: normal; one point: complaint of dizziness or walking difficulty, but no gait disorder in objective test; two points: gait instability, with ability to walk independently; three points: obvious abnormalities, with ability to walk only by virtue of assistance; and four points: inability to walk. (b) Cognition assessment: The Japanese iNPH scale (0–4 points) was applied to rate cognitive dysfunction, including zero point (normal); one point (complaint of memory decline and attention decline, but no memory and attention impairment in objective tests); two points (memory decline and distraction, but no temporal and spatial disorientation); three points (temporal and spatial disorientation, with communication ability); and four points (disorientation or complete inability to communicate). (c) Urinary incontinence assessment (0–4 points): It was evaluated as follows: zero point: normal; one point: frequent micturition or urgent micturition; two points: incident urinary incontinence (1–3 times/week or above, but <1 time/day); three points: frequent urinary incontinence (≥1 time/day); and four points: almost or complete loss of bladder function. The scoring standards of the mRS were set as follows: zero point: completely no symptoms; one point: with symptoms and ability to accomplish all the daily work and living, without apparent dysfunction; two points: mild disability, inability to complete all the activities before the illness, and ability to process daily affairs without other's help; three points: moderate disability, need for some help, and ability to walk independently; four points: severe disability, inability to walk independently, and need for other's help in daily living; five points: severe disability, confinement to bed, urinary and fecal incontinence, and complete dependence on other normal people in daily living; and six points: death. (2) As for the imaging assessment, the Evan's index, i.e., the ratio of the maximum distance between anterior horn of bilateral ventricles at the axial level to the maximum intracranial diameter at the same level (13, 14), was calculated according to preoperative cranial CT images of patients and the Evan's index > 0.3 indicated hydrocephalus. (3) In terms of the invasive assessment, cerebrospinal fluid tap test (tap test) is not only a method to observe the improvement in clinical symptoms after a certain amount of cerebrospinal fluid is released through lumbar puncture, but also an effective means of auxiliary diagnosis of iNPH. The tap test can be divided into single tap test and continuous lumbar cistern drainage test. In the former test, it is recommended that 30–50 ml of cerebrospinal fluid can be released every time and that the final pressure 0 of lumbar puncture can be set as the end point when the cerebrospinal fluid released cannot meet the above criteria. Moreover, cognitive disorder, gait disorder, and urinary dysfunction shall be assessed at least once within 8 and 24 h before and after tapping. The test will be conducted again within 72 h, if the results are negative (15–17). In continuous lumbar cistern drainage test, it is recommended that the cerebrospinal fluid can be released at 150–200 ml/day for 72 consecutive hours. In the case of progressive aggravation of clinical symptoms, it would be necessary to repeat the tap test at least 1 week later.

### Operative Methods

The operative indications included imaging results and clinical symptoms meeting the diagnostic criteria for iNPH, with secondary hydrocephalus and obstructive hydrocephalus excluded. Operating steps: After routine anesthesia, disinfection, and draping, the Kocher's point (at 2 cm before coronal suture and 3 cm lateral to the midline) was located, the scalp was cut open, and a hole was drilled to cut open the dura mater. Then, the frontal horn of right lateral ventricle was punctured using the ventricular end of shunt tube until clear cerebrospinal fluid flew out and the shunt tube was fixed at 6 cm. Next, a shunt valve was placed behind the ear, a subcutaneous tunnel was made along the neck, below the clavicle and beside the sternum, and the abdominal wall was cut open at 2 cm right below the xiphoid. Subsequently, the abdominal end of shunt tube was inserted for 10–15 cm; the distal ventricular end of the shunt tube was guided to the incision at the parietal tuber and then connected to the abdominal end. Later, the distal abdominal end of shunt tube was guided to the abdominal incision, a trocar for abdominal puncture was inserted, and the abdominal end of shunt tube was guided to the abdominal cavity. Medtronic adjustable shunt tube was used during operation and the cerebrospinal fluid shunting kits of the same model (Medtronic PS Medical Strata^@^11, Medtronic Incorporation) were used as the shunting devices, including antisiphon device, reservoir, and magnetic pressure-regulating valve. During operation, the initial pressure of shunt pump was determined by subtracting 20–30 mm H_2_O (1 mm H_2_O = 0.0098 kPa) from preoperative value of cerebrospinal fluid pressure measured by the tap test.

### Post-Operative Follow-Up

All the patients were followed-up by means of telephonic interview after operation and the clinical system score and overall living ability were evaluated using the iNPHGS and mRS, respectively. The improvement of clinical symptoms after cerebrospinal fluid shunt was defined as the mRS score decline by one point or above. The marked improvement of clinical symptoms was defined as the mRS score decline by two points or above.

### Statistical Analysis

Statistical Product and Service Solutions (SPSS) version 24.0 software (IBM Incorporation, Armonk, New York, USA) was employed to analyze the data. The measurement data in line with normal distribution were presented as mean ± SD ($\bar{\chi }$ ± s), the skewed data were expressed by median (range), and the paired samples *t*-test was performed to compare the differences in clinical symptoms before and after cerebrospinal fluid shunt among all the patients. Furthermore, related factors affecting the operative effects in the ineffective group, the effective group, and the markedly effective group of clinical treatment were analyzed through the chi-squared test and one-way ANOVA. The enumeration data were presented as absolute value or ratio. *P* < 0.05 suggested that the difference was statistically significant.

## Results

### Post-Operative Efficacy

Through comparing the evaluation indicators of clinical symptoms of patients with iNPH before and after cerebrospinal fluid shunt, it was found that there were different degrees of clinical manifestations among 65 patients with iNPH. The average pressure of cerebrospinal fluid was 127.2 ± 38.9 mm H_2_O the average number of cells was 127.2 ± 38.9 × 10^6^/L, and the average content of proteins was 0.59 ± 0.69 g/L. In addition, 61 (93.8%), 51 (78.5%), and 50 (76.9%) patients had gait disorder, cognitive disorder, and urinary incontinence disorder, respectively. There were 32 (49.2%) cases of typical Hakim's triad, 46 (70.8%) cases of gait disorder and cognitive disorder, 46 (70.8%) cases of gait disorder and urination disorder, 36 (55.4%) cases of cognitive disorder and urination disorder, and 0 cases of cognitive disorder, gait disorder, or urination disorder alone. All the 65 patients were followed up for 2–77 months, with an average of 48.7 ± 15.3 months and a median of 48 months. The duration from onset to operation was 6–156 months and 21.3 ± 26.3 months on average, with a median of 12 months.

Among 65 patients with iNPH, the preoperative mRS score was 5, 4, 3, 2, 1, and 0 points in 1 (1.5%), 15 (23.1%), 30 (46.2%), 15 (23.1%), 3 (4.6%), and 1 (1.5%) patients, respectively, and its mean value was 2.89 ± 0.92 points. As for the preoperative iNPHGS score, urinary incontinence was scored 4, 3, 2, 1, and 0 points in 2 (3.1%), 20 (30.8%), 21 (32.3%), 7 (10.8%), and 15 (23.1%) patients, respectively. Besides, 6 (9.2%), 32 (49.2%), 22 (33.9%), 1 (1.5%), and 4 (6.2%) patients had a gait disorder score of 4, 3, 2, 1, and 0 points, respectively. Finally, 10 (15.4%), 18 (27.7%), 23 (35.4%), and 14 (21.5%) patients were 4, 3, 2, and 0 points, respectively, regarding cognitive disorder.

After operation, the mRS score was 4, 3, 2, 1, and 0 points in 0, 6 (9.2%), 11 (16.9%), 37 (56.9%), and 11 (16.9%) patients with iNPH, respectively, and its mean value was 1.18 ± 0.83 points (paired samples *t*-test, *P* < 0.05). The postoperative urinary incontinence was scored 3, 2, 1, and 0 points in 1 (1.5%), 10 (15.4%), 25 (38.5%), and 29 (44.6%) patients, respectively. The gait disorder score was 3, 2, 1, and 0 points in 6 (9.2%), 11 (16.9%), 33 (50.8%), and 15 (23.1%) patients, respectively. Finally, 4 (6.2%), 21 (32.3%), 19 (29.2%), and 21 (32.3%) patients were given 3, 2, 1, and 0 points, respectively, regarding cognitive disorder.

According to the efficacy evaluation results after cerebrospinal fluid shunt, 57 patients with gait disorder (57/65, 87.7%) had improved gait function, 40 patients with cognitive dysfunction (40/65, 61.5%) had manifested ameliorated cognitive function, and 42 patients with urinary dysfunction (42/65, 64.6%) had exhibited improved symptoms of frequent micturition, urgent micturition, or urinary incontinence. The median mRS and iNPHGS scores during follow-up were statistically significantly different from those before operation [1 (0–4) points vs. 3 (0–4) points and 3 (0–12) points vs. 6 (0–12) points] (*P* < 0.05). On the premise that prognosis improvement is defined as the mRS score decline ≥ 1 point after operation compared with that before operation and marked prognosis improvement is defined as the mRS score decline ≥ 2 points after operation compared with that before operation, the patients were assigned into the non-improvement group (*n* = 8, 12.3%), the improvement group (*n* = 16, 24.6%), and the marked improvement group (*n* = 41, 63.1%) and the total effective rate of the VP shunt was 87.7%.

The mRS and iNPHGS scores were improved to different extent after cerebrospinal fluid shunt in contrast with those before operation (*P* < 0.05) and the symptoms were distinctly ameliorated after operation compared with those before operation, showing statistically significant differences. The changes in the mRS and iNPHGS scores before and after operation are given in Table 1.

**Table 1:** Comparisons of evaluation indicators of clinical symptoms of patients with iNPH before and after cerebrospinal fluid shunt.

**Time**	***n***	**mRS**	**iNPHGS**			
			**Cognitive disorder**	**Walking disorder**	**Urination disorder**	**Total score**
Before operation	65	2.89 ± 0.92	2.15 ± 1.33	2.54 ± 0.92	1.80 ± 1.20	6.49 ± 2.30
After operation	65	1.18 ± 0.83	1.12 ± 0.94	1.12 ± 0.88	0.74 ± 0.78	2.98 ± 1.96
Z		−6.67	−5.63	−6.55	−5.35	−6.73
*P*		<0.001	<0.001	<0.001	<0.001	<0.001

*Paired-samples t-test; iNPH, idiopathic normal-pressure hydrocephalus; mRS, Modified Rankin Scale; iNPHGS, idiopathic normal-pressure hydrocephalus grading scale*.

### Application of Invasive Examination (Tap Test) in Operative Effect Assessment

Before operation, four patients were subjected to lumbar cistern drainage test, while the remaining 61 patients received tap test and 20–50 ml of cerebrospinal fluid was obtained on average. For the patients with positive tap test results, the clinical manifestations such as cognitive, walking, and urination disorders were relieved in varying degrees after the VP shunt and the differences were statistically significant (*P* < 0.01). Besides, seven patients, who had negative tap test results (the symptoms were not notably alleviated following the tap test) and were diagnosed with iNPH based on the diagnostic criteria, continued to receive the VP shunt with the approval of themselves and their families. No statistical differences in the scores of urination and cognitive disorders were observed before and after treatment, but the score of walking disorder had a statistically significant difference after treatment (*P* = 0.045 < 0.05). These results suggest that active operative treatment for patients diagnosed with iNPH is of positive significance for the improvement of walking disorder, in spite of negative tap test results (Table 2).

**Table 2:** Comparisons of symptoms between the positive and negative tap test groups using Japanese iNPH grading scale (iNPHGS).

		**Cognitive disorder score**	**Walking disorder score**	**Urination disorder score**	**Total iNPHGS score**
Positive tap test group (*n* = 58)	Before treatment	2.26 ± 1.31	2.52 ± 0.94	1.86 ± 1.21	6.64 ± 2.37
	After treatment	1.14 ± 0.84	1.03 ± 0.84	0.72 ± 0.79	2.90 ± 2.02
	Z	−5.483	−6.302	−5.200	−6.507
	*P*	<0.001	<0.001	<0.001	<0.001
Negative tap test group (*n* = 7)	Before treatment	1.29 ± 1.25	2.71 ± 0.76	1.29 ± 1.11	5.29 ± 0.95
	After treatment	1.00 ± 1.16	1.86 ± 0.90	0.86 ± 0.69	3.71 ± 1.11
	t/z	1.549	2.521	1.441	−1.841
	*P*	0.172	0.045	0.200	0.066

*Paired-samples t-test*.

### Analysis of Related Factors Affecting the Effect of the VP Shunt

The comparison among the three groups indicated that the marked improvement group had a shorter onset time than the improvement group and the non-improvement group, but the difference was not statistically significant. However, statistically significant differences were found in complication with hypertension (*P* = 0.034 < 0.05) and clinical manifestation walking disorder (*P* < 0.05). The difference in the preoperative mRS score was statistically significant (*P* < 0.05), while the gender, age, preoperative lumbar puncture pressure, and postoperative complications showed no statistically significant differences among the three groups. The above findings imply that the patients complicated with hypertension and those complicated with walking and urination disorders can benefit greatly from the VP shunt and the higher preoperative mRS score may result in greater benefits after operation, indicating that the VP shunt is more beneficial to patients with more apparent symptoms (Table 3).

**Table 3:** Analysis of related factors affecting the effect of the ventriculoperitoneal (VP) shunt.

	**Total**	**Non-improvement group**	**Improvement group**	**Marked improvement group**	***χ^2^***	***P***
Male	39	5	8	26	0.963	0.614
Female	26	3	8	15		
Age (years old)	61.8 ± 11.8	51.4 ± 17.9	63.3 ± 10.2	63.3 ± 10.2	2.442	0.295
Preoperative mRS score	2.89 ± 0.92	1.75 ± 1.04	2.75 ± 0.93	3.17 ± 0.70	13.011	0.001
Preoperative lumbar puncture pressure	127.2 ± 38.9	125.6 ± 26.7	136.0 ± 43.2	124.2 ± 39.6	0.335	0.846
Onset time (month)	21.3 ± 26.3	25.1 ± 27.7	32.3 ± 42.7	17.6 ± 16.2	0.133	0.936
**Comorbidity**						
Hypertension (*n*)	22 ± 65	0 ± 8	4 ± 16	18 ± 41	6.525	0.034
Coronary heart disease (*n*)	6 ± 65	0 ± 8	0 ± 16	6 ± 41	2.713	0.224
Diabetes (*n*)	9 ± 65	0 ± 8	1 ± 16	8 ± 41	2.266	0.315
Cerebral infarct (*n*)	10 ± 65	0 ± 8	3 ± 16	7 ± 41	1.320	0.683
Epilepsy (*n*)	3 ± 65	0 ± 8	1 ± 16	2 ± 41	0.585	1.000
Cognitive and walking disorders	46 ± 65	5 ± 8	10 ± 16	31 ± 41	1.482	0.441
Cognitive and urination disorders	36 ± 65	4 ± 8	10 ± 16	22 ± 41	0.541	0.815
Walking and urination disorders	46 ± 65	3 ± 8	14 ± 16	29 ± 41	5.979	0.042
**Complication**						
Luminal blockage (*n*)	7 ± 65	3 ± 8	1 ± 16	3 ± 41	5.196	0.052
Abdominal infection (*n*)	3 ± 65	1 ± 8	1 ± 16	1 ± 41	2.417	0.305
Epileptic seizure (*n*)	1 ± 65	0 ± 8	0 ± 16	1 ± 41	1.234	1.000
Sudural hematoma (*n*)	1 ± 65	0 ± 8	1 ± 16	0 ± 41	3.116	0.369

*Chi-square test and one-way analysis of variance*.

### Post-Operative Complications

There were 12 (18.4%) cases of complications after operation, mainly including 7 (10.8%) cases of luminal blockage, 3 (4.6%) cases of abdominal infection, 1 (1.5%) case of subdural hematoma, and 1 (1.5%) case of epileptic seizure. Moreover, five out of 12 (7.7%) patients manifested ameliorated symptoms after replacing shunt tube and receiving anti-infection treatment, but 3 (4.6%) patients showed no alleviation following pressure adjustment due to advanced age and multiple complications. A total of 6 (9.2%) patients died of advanced age, pulmonary infection, heart failure, lung cancer, and myocardial infarction during follow-up (within 3–5 years after operation), and only 1 (1.5%) case of sudden death occurred within 2 weeks after operation, which was attributed to suspected pulmonary embolism. In addition, it was found through more than 5 years of follow-up after operation that 12 out of 23 (52.2%) patients had a good 5-year operative effect, 1 (4.3%) patient had been confined to bed due to advanced age and pulmonary infection, and 1 (4.3%) patient died of pulmonary infection and heart failure.

## Discussion

### Operative Effect

It has been reported in foreign literature that the symptom improvement rate of patients with iNPH is 69–84% at 1 year after shunt (18) and 61–85% at 3 years after shunt (19). In this retrospective study, the follow-up was classified as short-term follow-up (1–3 years after operation), medium-term follow-up (3–5 years after operation), and long-term follow-up (over 5 years after operation) according to the operation time of patients. The patients followed-up for 3–5 and over 5 years after operation were routinely inquired about the disease recovery in 1–3 and 3–5 years after operation. The results manifested that among 65 patients, 11, 31, and 23 patients were followed-up within 1–3 (inclusive), 3–5, and beyond 5 (inclusive) years after operation, respectively. It was discovered during follow-up that 8 (12.3%) patients showed no obvious changes, the effective rate of operation was 87.7%, and the symptom of 1 (1.5%) patient exacerbated again after 2 years, without recovery following pressure adjustment. With regard to the improvement of clinical manifestations, gait function was ameliorated most significantly, with an improvement rate of 87.7%, followed by urinary dysfunction (improvement rate: 64.6%) and cognitive decline (improvement rate: 61.5%). Moreover, the follow-up results revealed that patients with negative tap test results were eligible for the active VP shunt and, in particular, their walking disorder was relieved remarkably after operation, suggesting that active operative treatments can be considered on the basis of patients' clinical manifestations and imaging examinations as well as with the agreement of patients and their families, although the tap test results are negative. It is also indirectly indicated that the tap test has a fairly high positive predictive value but slightly low sensitivity, meaning that the tap test cannot be regarded as the only screening indicator for iNPH operation. In current treatment of iNPH, excessive emphasis is put on the definite diagnosis of patients. Besides, differential diagnosis is overemphasized and no positive surgical treatments are adopted for patients without typical clinical symptoms. Furthermore, among 23 patients followed-up at more than 5 years after operation, the operative effect remained good for 5 years in 12 (52.1%) patients and there was 1 (4.3%) case of confinement to bed due to advanced age and pulmonary infection and 1 (4.3%) case of death from pulmonary infection and heart failure. Some researchers have reported that the 5-year mortality rate of untreated patients with suspected iNPH is up to 87.5% and iNPH can increase the risks of irreversible dementia and early death (20). Additionally, patients with a disease history < 6 months have a higher symptom improvement rate after treatment than those with a disease history > 2–3 years or complicated with dementia (21). Therefore, shunt is the only effective treatment method for iNPH, reflecting its importance in early diagnosis and treatment of iNPH.

### Post-Operative Complications

Infection, subdural hematocele and effusion, excessive shunt, epilepsy, shunt tube-related complications (e.g., blockage and leakage), and abdominal incision pain are the major complications after the VP shunt. Ma et al. (22) conducted follow-up for 102 patients with iNPH after the VP shunt and 40% of patients had the aforementioned complications after operation. In the study of Larsson et al. (23), 176 patients with iNPH were followed-up after the VP shunt and it was discovered that headache (36.1%), abdominal incision pain (20%), and epilepsy (4.5%) are the most common postoperative complications. In this study, complications occurred in 12 (18.4%) patients after operation, dominated by 7 (10.8%) cases of luminal blockage, 3 (4.6%) cases of abdominal infection, 1 (1.5%) case of sudural hematoma, and 1 (1.5%) case of epileptic seizure. Moreover, the symptoms of 5 (7.6%) patients were ameliorated after shunt tube replacement and anti-infection treatment, but the symptoms were not alleviated in 3 (4.6%) patients after pressure adjustment due to advanced age and multiple complications. Furthermore, 6 (9.2%) patients died of advanced age, pulmonary infection, heart failure, lung cancer, and myocardial infarction during follow-up (within 3–5 years after operation) and only 1 (1.5%) case of sudden death occurred within 2 weeks after operation, which was attributed to suspected pulmonary embolism. Hence, it is recommended that patients with iNPH can be reexamined by CT regularly in addition to active prevention of infection and timely revisit in the case of disease change and prompt understanding of intracerebral shunt pressure is equally crucial for reducing postoperative complications. Additionally, it was observed through long-term follow-up that cognitive disorder recovered slowly, while gait disorder was restored best, followed by urinary incontinence.

### Comorbidities

Generally, patients with iNPH are complicated with multiple diseases such as cerebrovascular disease, Parkinson's, Alzheimer's disease, and vascular dementia. Boon et al. (24) argued that cerebrovascular disease is the most common comorbidity of patients with iNPH and the incidence rate of stroke is as high as 45% in all the patients with iNPH. Koivisto et al. (25) enrolled 146 patients with iNPH, of whom 18 (12.3%) patients were diagnosed with Alzheimer's disease and 8 (5.5%) patients were diagnosed with vascular dementia at the same time. Besides, the research results of Allali et al. (26) demonstrated that the existence of comorbidities can influence the improvement of gait of patients with iNPH after tap test, so the possibility of other comorbidities, especially Alzheimer's disease, needs to be considered for patients with iNPH with poor gait improvement after tap test. Therefore, the influences of some comorbidities on gait, urinary incontinence, and cognitive functions following shunt should be investigated in the future prospective studies, so as to better understand the long-term prognosis of patients with iNPH. In this study, 34 out of 65 (52.3%) patients had complications, including primary hypertension (*n* = 22, 33.8%), stroke history (*n* = 10, 15.4%), diabetes history (*n* = 9, 13.8%), coronary heart disease history (*n* = 6, 9.2%), Parkinson's disease history (*n* = 1, 1.5%), and epilepsy (*n* = 3, 4.6%).

### Analysis of Related Factors Affecting the Effect of the VP Shunt

The VP shunt is the most frequently used therapeutic method for patients with iNPH at present, but there are still considerable controversies over the diagnosis and selection of patients for operation. The common prediction methods for iNPH in clinic include tap test, cerebrospinal fluid perfusion test, continuous intracranial pressure tracing and monitoring, and specific imaging methods (diffusion-weighted imaging, magnetic resonance three-dimensional-fast spin-echo sequence-drive pulse imaging, magnetic resonance spectroscopy, etc.). However, none of these methods can accurately predict the operative effect and are difficult to implement in clinic. Hence, new prediction methods, including inflammatory mediator (27) and proteomics (28), will be the main directions of research in the future. In this study, the mean mRS score of patients with iNPH was decreased significantly after operation compared with those before operation and the differences were statistically significant. The analysis results of related factors illustrated that patients complicated with hypertension and those complicated with walking and urination disorders benefited greatly, suggesting that the VP shunt is more beneficial to patients with more apparent symptoms. Some studies have denoted that the operative effect will be more prominent if the onset time (from the occurrence of clinical symptoms to the time of operation) is shorter, implying that patients with a shorter onset time are more likely to benefit from the VP shunt. According to the results in this study, the onset time was shortened in the marked improvement group in comparison with that in the improvement group and the non-improvement group, but the difference was not statistically significant. In the future, clinical studies with larger sample sizes will be conducted to further seek the influencing factors for the efficacy of the VP shunt.

As the clinical diagnosis and treatment level of iNPH is elevated, it has been corroborated that surgery is an efficacious therapeutic measure for iNPH and early operation is capable of remarkably ameliorate the disease condition and prognosis of patients. Therefore, patients diagnosed with iNPH should receive operation as early as possible after the clinical diagnosis is fully evaluated. Generally, the symptoms of iNPH can be mitigated by removing excess cerebrospinal fluid *via* operation. Currently, increasingly more attention has been paid to iNPH by both the neurology and neurosurgery and the early diagnosis and treatment of iNPH, a reversible cause of dementia, is conducive to improving patients' quality of life. In addition, the effect of surgery is good although the pathogenesis of iNPH has not been clarified.

## Conclusion

In conclusion, the results of this study demonstrated that the shunt is able to improve the gait, cognition, and urinary dysfunctions of patients with iNPH to different extent, including the long-term symptoms. The gait disorder is relieved more distinctly and constantly after the shunt compared with other symptoms. The shunt leads to a low incidence rate of complications and guarantees successful treatment for 70% patients in several years after operation. Furthermore, the efficacy remains preferable in most patients during follow-up at more than 5 years after operation, the quality of life of patients is notably improved, and the detailed diagnostic procedures and accurate selection of patients are the key factors for satisfying postoperative results of the operated patients. Finally, regular clinical follow-up is the guarantee of long-term effectiveness of the operation. The deficiency of this study is that the single-center retrospective study may have certain bias of data, which needs to be further verified by prospective controlled study. Moreover, the results of this study shall be demonstrated through large-sample multicenter trials on iNPH.

## Data Availability Statement

The original contributions presented in the study are included in the article/supplementary material, further inquiries can be directed to the corresponding author.

## Ethics Statement

The studies involving human participants were reviewed and approved by the Ethics Committee of Tianjin Huanhu Hospital. The patients/participants provided their written informed consent to participate in this study.

## Author Contributions

RS, HN, and LC designed the study, performed the experiments, and prepared the manuscript. NR and XX collected the data. XC, GL, and XL analyzed the data. All authors read and approved the final version of the manuscript.

## Funding

This study was supported by Key Research and Development Program of Jining City (Study on the role and mechanism of TYROBP gene in Tourette syndrome; Project no. 2021YXNS082).

## Conflict of Interest

The authors declare that the research was conducted in the absence of any commercial or financial relationships that could be construed as a potential conflict of interest.

## Publisher's Note

All claims expressed in this article are solely those of the authors and do not necessarily represent those of their affiliated organizations, or those of the publisher, the editors and the reviewers. Any product that may be evaluated in this article, or claim that may be made by its manufacturer, is not guaranteed or endorsed by the publisher.
